# Post-translational modifications of mitochondrion-related proteins: integrative orchestrators of cell fate networks in cardiovascular disease

**DOI:** 10.1186/s12964-026-03002-y

**Published:** 2026-06-13

**Authors:** Lei Liu, Saiteng Wu, Lei Zhang, Li Zhong, Rui Guo

**Affiliations:** 1 College of Life Sciences, Institute of Life Science and Green Development, Hebei University, Baoding, 071002 China; 2 Baoding Key Laboratory of Cancer and Aging, Hebei University, Baoding, 071002 China; 3 College of Osteopathic Medicine of the Pacific, Western University of Health Sciences, Pomona, CA 91766 USA; 4 The Key Laboratory of Zoological Systematics and Application, College of Life Sciences, Hebei University, Baoding, 071002 China

**Keywords:** Cardiovascular disease, Mmitochondrial quality control, Post-translational modifications, Cell death, Dynamic equilibrium.

## Abstract

Cardiovascular disease remains the leading cause of global mortality, with mitochondrial dysfunction playing a central pathogenic role. Post-translational modifications act as fundamental regulators of mitochondrial quality control. Yet, how mitochondrial post-translational modifications integrate stress signals to direct cell fate among diverse regulated cell death pathways in cardiovascular disease remains incompletely understood. This review proposes a conceptual framework in which mitochondrial post-translational modifications act as the master conductors of an integrated network linking mitochondrial homeostasis to cellular demise. We first outline the pivotal roles of mitochondrial quality control in cardiovascular disease and detail their precise mechanisms governed by mitochondrial post-translational modifications over each process. We then delineate how mitochondrial post-translational modifications critically regulate the initiation and execution of apoptosis, necroptosis, pyroptosis, ferroptosis, and cuproptosis, evaluating their distinct contributions to cardiovascular pathophysiology. Furthermore, we highlight the extensive crosstalk and convergence among these death modalities at the mitochondrial level, emphasizing the role of mitochondrial post-translational modification signatures in amplifying death signals or triggering modality switching. By synthesizing recent discoveries, this work connects dynamic protein-level modifications to cell fate outcomes, offering a theoretical basis for future therapeutic strategies aimed at rebalancing the network of mitochondrial post-translational modifications to combat heart failure and other cardiovascular diseases.

**Supplementary Information:**

The online version contains supplementary material available at 10.1186/s12964-026-03002-y.

## Introduction

Cardiovascular diseases (CVD) are the foremost cause of mortality worldwide, accounting for over 30% of all deaths, and their global burden is rising alongside population aging [1, 2]. Mitochondria, present in all eukaryotic cells, are central orchestrators of cardiovascular physiology and pathology [3]. Under physiological conditions, the abundant mitochondria in cardiomyocytes not only continuously generate ample ATP via oxidative phosphorylation to power cardiac contractions [4], but also regulate crucial cellular processes like calcium homeostasis and reactive oxygen species (ROS)-derived signaling [5]. Conversely, under pathological stress, mitochondrial dysfunction compromises ATP production, exacerbates oxidative stress, and induces calcium overload [6], thereby promoting the pathogenesis of CVD such as atherosclerosis (AS) [7], myocardial ischemia-reperfusion injury (MIRI) [8], and heart failure [9].

Mitochondrial quality control (MQC) is a multi-layered protective system that preserves mitochondrial integrity, function, and turnover. Maintaining mitochondrial homeostasis through robust MQC is therefore essential for cardiovascular health. In recent years, the discovery of novel regulated cell death (RCD) modalities, such as necroptosis, pyroptosis, and ferroptosis, has greatly expanded our understanding of cellular demise [10]. Mitochondria act as a critical hub for these processes [11]: they not only serve as integrators of apoptotic signals by releasing cytochrome C and other factors to initiate apoptosis [12], but also regulate the RIPK1-RIPK3-MLKL pathway during necroptosis [13], promote inflammasome activation in pyroptosis [14], and drive lipid peroxidation in ferroptosis [15]. Importantly, in chronic CVD models, the absolute number of terminally dying cells detected in vivo is often low. However, even sub-lethal activation of RCD signaling pathways can profoundly influence disease progression by promoting inflammation, fibrosis, and contractile dysfunction [16, 17]. Consequently, the functional status of mitochondria and the capacity of MQC are pivotal in determining the mode of cell death, profoundly influencing the onset and progression of CVD.

Post-translational modifications (PTMs) are reversible, site-specific chemical alterations that act as molecular switches, regulating protein activity, localization, stability, and interactions [18]. The regulatory power of PTMs lies in their reversibility and their ability to occur on timescales ranging from seconds (e.g., phosphorylation) to hours (e.g., certain forms of acetylation or ubiquitination), allowing for both rapid adaptation and long-term reprogramming of cellular states [19, 20]. In mitochondria, PTMs precisely control all aspects of MQC, and thereby influence CVD progression [21]. Specifically, PTMs of mitochondrion-related proteins (mtPTMs) orchestrate mitochondrial protein function and transduce diverse intra- and extracellular signals into coordinated responses of mitochondrial remodeling. Thus, they serve as a critical nexus that links mitochondrial status to cell fate decisions.

This review systematically explores the mechanisms by which mtPTMs regulate MQC and influence the selection among RCD pathways in CVD. By synthesizing current evidence and delineating these regulatory networks, we aim to establish a coherent theoretical framework to guide future research and therapeutic strategies targeting mtPTMs for CVD treatment.

## Mitochondrial quality control: an integrated network governing organelle homeostasis

**Fig. 1:**
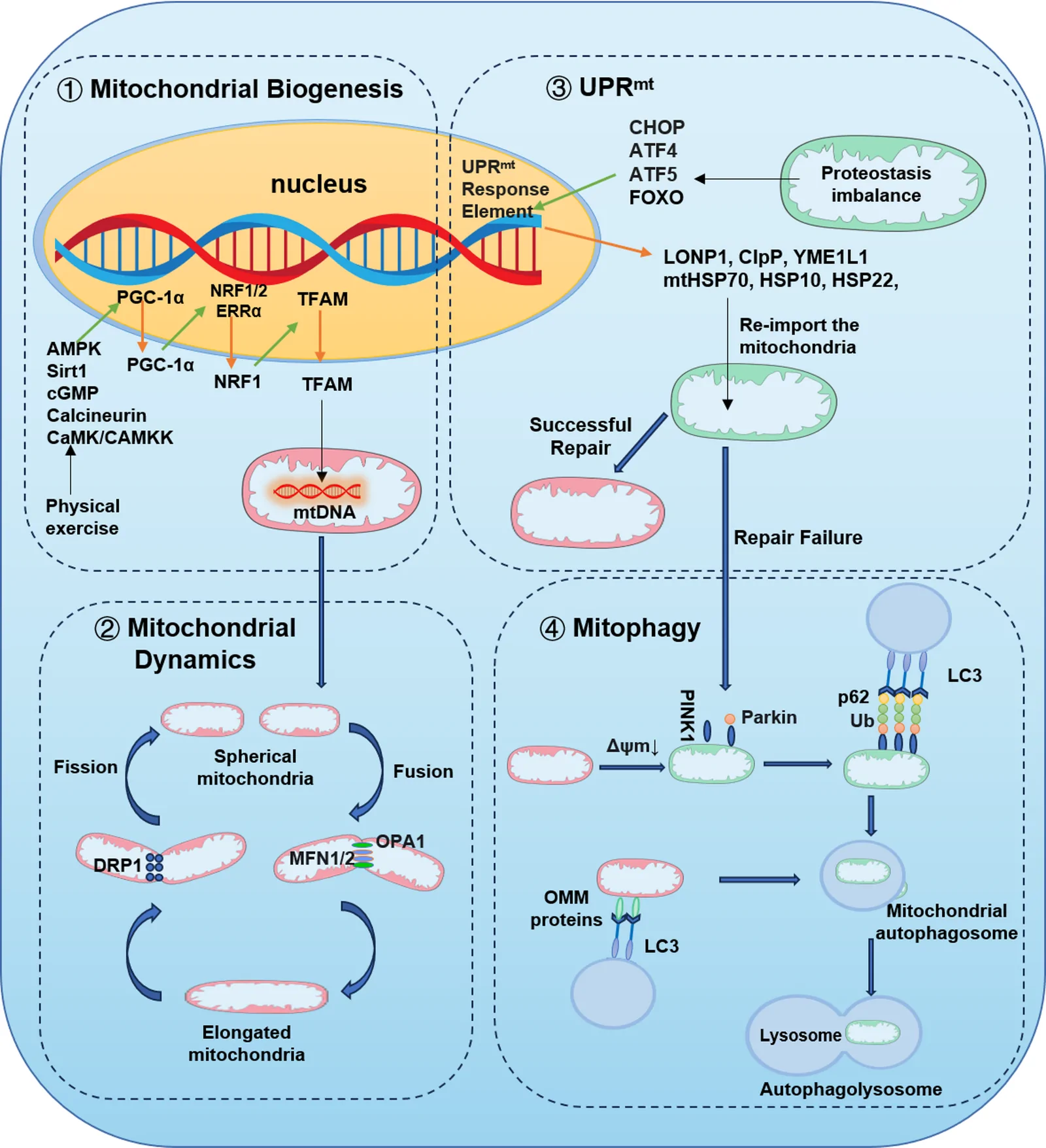
An integrated network of MQC mechanisms. Mitochondrial homeostasis is maintained through four core, interconnected processes. Biogenesis generates new mitochondria via the PGC-1α/NRF1/TFAM pathway to replenish the network. Dynamics remodels this network continuously through fission and fusion, with DRP1 and MFN1/2/OPA1 as the key mediators facilitating content mixing and distribution, respectively. When mitochondrial proteostasis is challenged, key transcription factors, including ATF5, ATF4, CHOP, and FOXO, translocate to the nucleus, bind UPRᵐᵗ response elements, and upregulate mitochondrial chaperones (mtHSP70, HSP10, HSP22) and proteases (LONP1, CLPP, YME1L1) to alleviate stress. Finally, mitophagy identifies and clears terminally damaged mitochondria through the PINK1/Parkin-dependent pathway or via specific outer mitochondrial membrane (OMM) proteins. These mechanisms collectively sustain a healthy and functional mitochondrial pool

MQC is a coordinated system that safeguards mitochondrial integrity via four dynamically interlinked processes: biogenesis, dynamics, mitophagy, and the mitochondrial unfolded protein response (UPRᵐᵗ). Dysregulation of this system is a pivotal event in CVD pathogenesis [22]. As illustrated in Fig. 1, these mechanisms function as an integrated network to maintain organellar homeostasis. This figure depicts the four core processes—biogenesis, dynamics, mitophagy, and UPRᵐᵗ—as interconnected modules that collectively determine the health of the mitochondrial pool. The following section examines the role of each MQC component in CVD and the pathological consequences of its impairment.

### Mitochondrial biogenesis: generation of new mitochondria and its dysregulation in CVD

Mitochondria occupy 20–30% of the cardiomyocyte volume [23]. As dynamic organelles, they continuously adapt to tissue metabolic demands, a process facilitated by increasing their numbers through mitochondrial biogenesis in response to elevated energy requirements [24]. This biogenesis is regulated by an energy-sensing cascade involving factors such as AMPK, SIRT1, calcineurin, CaMK/CAMKK, and cGMP. PGC-1α is a central mediator that activates downstream transcription factors and upregulates TFAM expression, subsequently promoting mitochondrial DNA (mtDNA) replication and transcription [25–28].

Disruption of mitochondrial biogenesis contributes to ATP deficiency and metabolic dysregulation in CVD [29]. For example, in rodent models, nutrient excess coupled with reduced energy demand in obesity or insulin resistance suppresses mtDNA gene expression, which diminishes mitochondrial biogenesis and oxidative phosphorylation capacity, ultimately impairing myocardial function [30]. Cardiac mitochondrial dysfunction and impaired biogenesis have been observed in dyslipidemic mouse models [31]. Endothelial nitric oxide synthase (eNOS) also regulates mitochondrial biogenesis [32], and eNOS uncoupling can attenuate biogenesis and compromise cardiac function by downregulating PGC-1α in hypertensive mouse models [33].

### Mitochondrial dynamics: balancing fission and fusion in cardiac health and disease

Mitochondrial dynamics refers to a highly regulated process that maintains mitochondrial morphological and functional integrity through a homeostatic equilibrium between fission and fusion. Fission is mediated by the recruitment of dynamin-related protein 1 (DRP1) to the outer mitochondrial membrane (OMM) via receptors such as FIS1 and MFF [34], facilitating segregation of damaged mitochondria [35]. Fusion involves OMM fusion by MFN1/2 and inner membrane fusion by OPA1, enabling content exchange and energy equilibration [36, 37]. Disruption of this balance leads to abnormal morphology, which can exacerbate oxidative stress and cell death.

Mitochondrial dynamics is implicated in CVD. In human patients with heart failure (HF), excessive fission coupled with impaired fusion is a common feature that is associated with poor prognosis [38]. Consistent with this, murine studies demonstrate that DEAD-box helicase 17 (DDX17) attenuates DRP1-mediated fission and preserves cardiac function by targeting this pathway [39]. Similarly, in a murine model of HF with preserved ejection fraction (HFpEF), S100A9 inhibition alleviates pathological fission and oxidative stress via DRP1 regulation [40]. Conversely, in murine models of diet-induced obesity, genetic knockdown of DRP1 exacerbates cardiac dysfunction and remodeling [41], emphasizing its context-dependent function in CVD. This duality extends to vascular pathology, in a mouse model of AS, the DRP1 inhibitor Mdivi-1 attenuates disease progression by suppressing DRP1-dependent mitochondrial fission in vascular cells [42]. Paradoxically, melatonin suppresses AS not by promoting fusion, but by inhibiting OPA1-mediated fusion [43], further underscoring the disease-specific complexity of dynamics. Collectively, these findings indicate that maintenance of mitochondrial function depends not on simply promoting or inhibiting fission and fusion, but on reestablishing an optimal dynamic equilibrium under specific pathological conditions.

### Mitophagy: a double-edged sword in mitochondrial clearance and CVD pathogenesis

Mitophagy is a cargo-specific autophagic process that removes damaged mitochondria [44], essential for sustaining mitochondrial quantity and energy homeostasis [45]. Its role is highly context-dependent and exists on a spectrum. At baseline, physiological mitophagy constitutively removes aged or mildly damaged organelles to prevent ROS accumulation [46]. Under moderate stress such as early ischemia/reperfusion (I/R), adaptive mitophagy is upregulated to clear compromised mitochondria and is protective. However, severe or chronic stress can trigger maladaptive mitophagy, where excessive organelle clearance depletes the mitochondrial pool, impairs bioenergetics, and exacerbates cell death [47]. The threshold between these states is governed by stress intensity and duration.

Mitophagy is primarily governed by two mechanisms. The first is the ubiquitin (Ub)-dependent PINK1/Parkin pathway. Upon mitochondrial depolarization, PINK1 stabilizes on the OMM and recruits Parkin, which ubiquitinates mitochondrial proteins [48, 49]. These ubiquitinated proteins are recognized by p62 and bound to LC3, triggering engulfment by autophagosomes [50]. The second mechanism operates through Ub-independent, receptor-mediated pathways. OMM proteins such as BNIP3, NIX, and FUNDC1 harbor a conserved LC3-interacting region that enables direct binding to LC3 on autophagosomes to initiate mitophagy [51].

Mitophagy directly influences CVD onset and progression. Upon mitochondrial stress, PINK1 accumulates on the OMM and recruits autophagy adaptors such as NDP52 and optineurin (OPTN) to initiate mitophagy [52]. Its impairment causes dysfunctional mitochondrial accumulation and ROS overproduction to activate Nrf2 which then upregulates PINK1 and p62, forming a protective feedback loop in cardiomyocytes [53]. If this defense fails, BNIP3/NIX-dependent mitophagy is critical to attenuate ferroptosis by controlling mitochondrial ROS [54]. In a rat model of MIRI, disruption of the SIRT1-SIRT3 axis inhibits PINK1/Parkin signaling to promote ferroptosis and tissue injury [55]. On the other hand, FUNDC1 activation or AP39 treatment reduces pyroptosis and improves mitophagy, which alleviates doxorubicin-induced fibrosis [56]. Similarly, in mice, phosphodiesterase 4D inhibition enhances mitophagy and protects against cardiac hypertrophy and failure [57], while ULK1-mediated mitophagy opposes pressure overload-induced remodeling in mouse hearts [58]. In line with this, we previously showed in a mouse model that activating TFEB to restore mitophagy mitigates ethanol-induced cardiac damage [59].

### The mitochondrial UPRᵐᵗ: safeguarding proteostasis in the stressed heart

The UPRᵐᵗ is a conserved adaptive mechanism triggered by the loss of mitochondrial proteostasis [60]. Accumulation of unfolded proteins or stoichiometric imbalances in the electron transport chain activates a signaling cascade that culminates in the nucleus. This process is governed by the integrated stress response (ISR), under which kinases such as GCN2 and PERK phosphorylate eIF2α. This leads to a global attenuation of translation, while selectively promoting the translation of key transcription factors such as ATF4, ATF5, and CHOP [61, 62], which subsequently translocate to the nucleus and drive the expression of mitochondrial proteases (e.g., LONP1, CLPP) and chaperones (e.g., HSP60, HSP10) to restore proteostasis [63].

The UPRᵐᵗ is essential for maintaining mitochondrial function and supporting cell survival in CVD. In myocardial biopsies from patients with chronic ischemic and dilated cardiomyopathy, UPRᵐᵗ-related genes are broadly downregulated, indicating a collapse of MQC system in advanced human disease [64]. During acute myocardial ischemia in rodent models, UPRᵐᵗ is activated as an early adaptive response [65]. However, reperfusion can disrupt UPRᵐᵗ homeostasis, altering downstream signaling. Prolonged CHOP expression, in particular, may then shift the response from pro-survival toward pro-apoptotic pathways, promoting cardiomyocyte death [66, 67].

In summary, MQC preserves mitochondrial integrity and function through a coordinated system that integrates mitophagy, biogenesis, dynamics, and UPRᵐᵗ. Disruption of this equilibrium drives the progression of cardiovascular pathologies.

## Regulation of MQC by PTMs: a post-translational control hub in cardiovascular homeostasis and disease

PTMs act as fundamental molecular switches that dynamically regulate all components of the MQC system. It is useful to conceptualize these modifications within a regulatory hierarchy. Primary regulatory PTMs, such as AMPK-mediated phosphorylation of PGC-1α or SIRT3-mediated deacetylation of multiple targets, function as master switches that coordinate broad cellular programs [68]. Secondary/adaptive modifications, like the ubiquitination of a specific cargo receptor, execute precise, localized functions [69]. Furthermore, temporal dynamics are crucial: rapid, reversible phosphorylation cascades (seconds to minutes) enable swift adaptation to acute stress (e.g., I/R) [70], whereas more stable modifications such as acetylation or SUMOylation often underpin long-term metabolic reprogramming seen in chronic conditions (e.g., HF) [71]. Dysregulation of these PTM networks disrupts MQC homeostasis and triggers mitochondrial dysfunction, a central pathogenic mechanism in diverse CVDs. This section delineates how specific PTMs govern mitophagy, dynamics, biogenesis, and UPRᵐᵗ.

### The regulation of mitophagy by PTMs

**Fig. 2:**
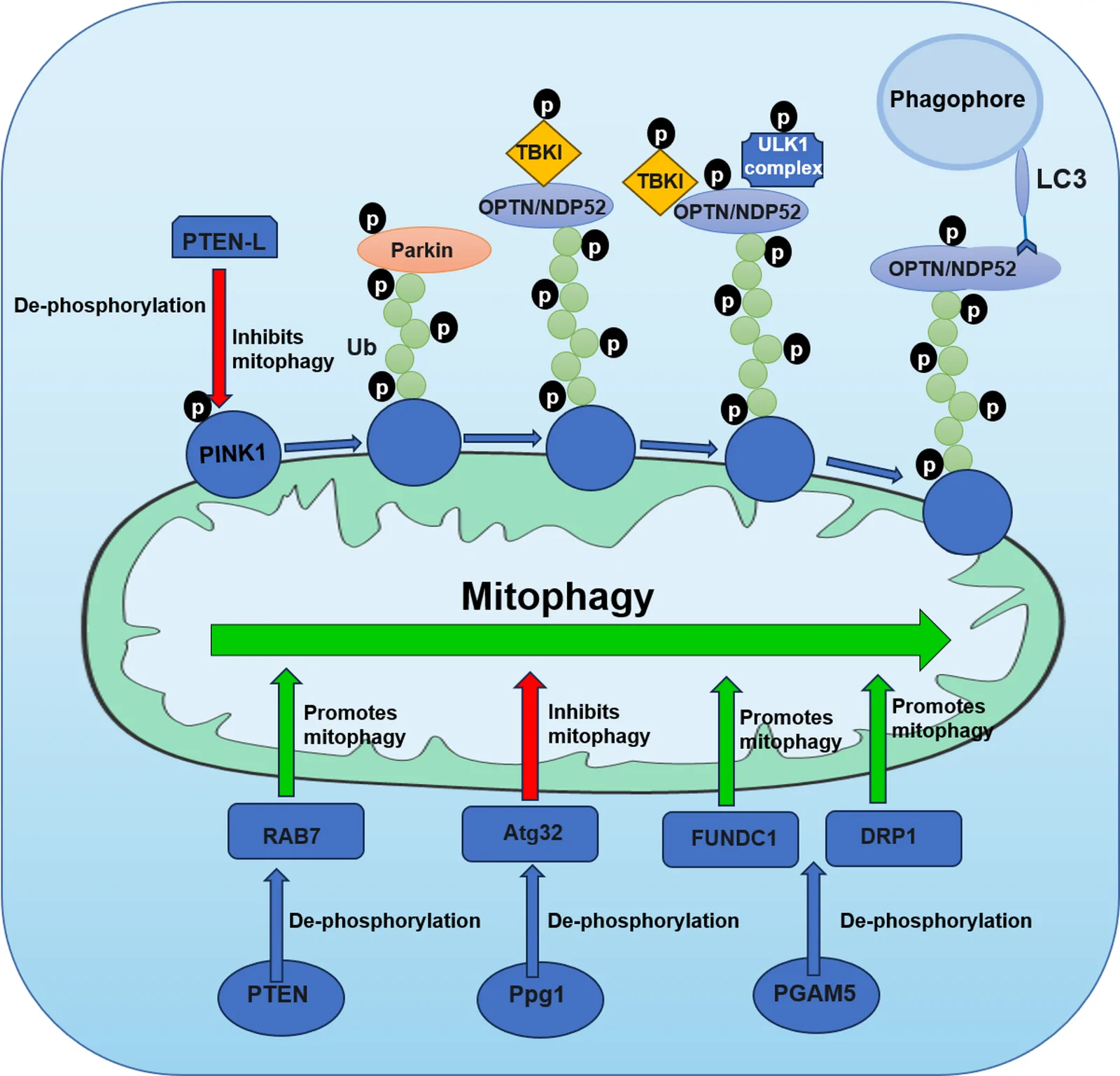
Phosphorylation and Dephosphorylation in Mitophagy Regulation. Triggered by mitochondrial depolarization, PINK1 stabilizes on the OMM and phosphorylates Ub and Parkin, serving as a critical upstream signal for mitophagy initiation. This cascade recruits primary mitophagy receptors such as OPTN and NDP52, along with TANK-binding kinase 1 (TBK1), which is subsequently activated on OMM. Activated TBK1 then phosphorylates these receptors to enhance their affinity for Ub chains, promoting further recruitment of receptors and TBK1 in a positive feedback mechanism that potentiates the mitophagic signal. The ULK1 complex is then recruited through these receptors, facilitating LC3 lipidation and initiating phagophore formation. Notably, PTEN-L has been shown to suppress both PINK1 phosphorylation and PINK1-mediated Ub phosphorylation. Meanwhile, phosphatase-mediated dephosphorylation events exert bidirectional control over mitophagy: PTEN promotes mitophagy by dephosphorylating RAB7, Ppg1 inhibits mitophagy by dephosphorylating Atg32, and PGAM5 promotes mitophagy by dephosphorylating FUNDC1 and DRP1

Mitophagy fidelity in CVD depends on PTMs, which precisely regulate clearance of damaged mitochondria via phosphorylation, ubiquitination, and acetylation of core factors [72]. Among these PTMs, phosphorylation plays a particularly pivotal role in cardiac pathophysiology, orchestrating a mitophagy pathway essential for cardiomyocyte homeostasis [61]. As illustrated in Fig. 2, this process is centrally governed by the PINK1-TBK1 phosphorylation cascade and counter-regulatory phosphatases in tuning the mitophagic response. The canonical pathway involves PINK1 stabilization on the OMM, phosphorylating Parkin and ubiquitin chains. This recruits autophagy receptors (e.g., OPTN, NDP52) and activates TBK1, initiating mitophagy [73]. TBK1 further phosphorylates receptors, creating a positive feedback loop.

The activity of this PINK1-Parkin axis must be precisely tuned. Its downregulation contributes to mitochondrial dysfunction in heart failure [74], while its excessive activation during I/R can exacerbate injury, indicating that mitophagy must operate within a precise threshold [47]. This paradigm of context-dependence extends to other key regulators. The mitochondrial phosphatase PGAM5 exemplifies such complexity. PGAM5 can promote mitophagy by dephosphorylating FUNDC1 [75], yet it exacerbates damage in doxorubicin-induced cardiotoxicity by disrupting mitochondrial dynamics [76]. Its role in cardiac I/R injury remains contentious, with studies supporting both a protective function via facilitating PINK1-dependent mitophagy [77] and a detrimental role primarily through driving necroptosis [78]. These divergent findings likely stem from critical differences in experimental models (ex vivo Langendorff perfusion vs. in vivo coronary ligation), the temporal phase of injury (acute early reperfusion vs. subacute late reperfusion), or the cellular context (cardiomyocyte-autonomous effects in conditional knockouts vs. systemic effects in global knockouts), highlighting the need for spatiotemporally resolved studies to clarify PGAM5’s function.

A separate layer of regulation involves the balance of ubiquitination and deubiquitination. E3 ligases such as MARCH5 and MUL1 work alongside Parkin to tag damaged mitochondria [79, 80], while deubiquitinating enzymes (DUBs) like USP30 remove these signals [81–83]. Despite showing promise in enhancing mitochondrial clearance in preclinical models of cardiac dysfunction, strategies targeting this balance, such as USP30 inhibition, have yet to demonstrate clinical translatability [84]. Moreover, acetylation intricately links mitophagy to cellular metabolic status. The NAD^+^-dependent deacetylase SIRT3 activates mitophagy by deacetylating PINK1 and Parkin, linking cellular energy status to MQC [85]. This SIRT3-mediated pathway is compromised in aging and metabolic syndrome, as demonstrated in rodent models of pulmonary hypertension associated with HFpEF, thereby contributing to cardiometabolic disease progression [86]. Additionally, stress-induced O-GlcNAcylation during simulated MIRI in vitro enhances SIRT3 activity, thus protecting cardiomyocytes [87]. In summary, cardiac mitophagy is finely tuned by a multi-layered PTM network that integrates metabolic, oxidative, and survival signals.

### The regulation of mitochondrial dynamics by PTMs

Mitochondrial dynamics are exquisitely regulated by PTMs. DRP1, the master regulator of mitochondrial fission, is a major hub for PTM integration. Its activity is controlled by a hierarchy of modifications. Phosphorylation at Ser616 (by CDK1/ERK) promotes fission, while phosphorylation at Ser637 (by PKA) inhibits it in multiple mammalian cell types and tissues [88, 89]. Consistently, in failing human hearts, decreased Ser616 phosphorylation of DRP1 in mitochondrial fractions is associated with impaired mitophagy and contributes to HF progression [90]. Pharmacological or genetic inhibition of DRP1 ameliorates pathology in mouse models of AS, HF and I/R injury [91–94]. Beyond phosphorylation, DRP1 is also regulated by acetylation at Lys642 (promoting fission), and this acetylation promotes fission under lipid overload in mouse hearts and rat cardiomyocytes, and SIRT1-mediated deacetylation may counteract this effect [95]. Lipid overload in obese mice and monkeys drives this acetylation, exacerbating cardiac dysfunction and suggesting therapeutic benefit from SIRT1 activators [96]. Furthermore, O-GlcNAcylation at Thr585/586 of DRP1 enhances its fission activity, contributing to mitochondrial dysfunction in rodent diabetic cardiomyopathy [97, 98]. SUMOylation stabilizes DRP1, and its reduction protects against sepsis-induced cardiomyopathy in mice [99, 100], whereas ubiquitination targets DRP1 for degradation, a mechanism exploited by the E3 ligase FBXL4 to suppress excessive fission in mouse models of HFpEF [101]. Similarly, in mouse models of diabetic cardiomyopathy, MAP4K4 promotes S-nitrosylation of DRP1 at Cys644, driving excessive fission [102]. Thus, a sequential hierarchy governs DRP1 regulation. Phosphorylation at Ser616/Ser637 controls its mitochondrial recruitment. Once localized, the degree and duration of its fission activity are modulated by acetylation, O-GlcNAcylation, SUMOylation, and ubiquitination, thus linking stress signals to sustained changes in cardiac mitochondrial dynamics.

Fusion proteins are equally regulated. Proteolytic cleavage of OPA1 by OMA1 promotes fission during cellular stress [103]. In mouse hearts, SIRT3 deficiency enhances OPA1 acetylation and cleavage, suppressing fusion in cardiac aging and pressure overload models [104, 105]. MFN1/2 phosphorylation inhibits mitochondrial fusion capacity and initiates mitophagy in cardiomyocytes [106], while ubiquitination precisely controls their stability and activity [107, 108].

Collectively, PTM-mediated control of mitochondrial dynamics-related proteins functions as a dynamic rheostat that governs mitochondrial morphology. In CVD, chronic pathological stimuli can impair this machinery regulator, rendering restoration of balanced PTM profiles a promising therapeutic strategy.

### The regulation of mitochondrial biogenesis by PTMs

Mitochondrial biogenesis is critical for renewing the mitochondrial pool in response to stress or increased demand. The master regulator PGC-1α functions as a signaling hub to integrate diverse physiological and pathological cues through a complex layer of PTMs at the molecular level. Specifically, PGC-1α contains an intrinsic repressive domain, whose inhibition is relieved upon phosphorylation at Thr262, Ser265, and Thr298 by p38 MAPK [109]. In contrast, phosphorylation of PGC-1α at Ser570 by Akt and by Cdc2-like kinase 2 within its SR domain suppresses PGC-1α coactivator function [110]. In the vasculature, this mechanism has been demonstrated to mediate angiotensin II-induced vascular smooth muscle cell hypertrophy and oxidative stress [111]. In the context of diabetic cardiomyopathy, this phosphorylation event is hypothesized to underlie the impaired mitochondrial biogenesis observed in insulin-resistant states. However, direct in vivo evidence in cardiac tissue is still lacking. Meanwhile, AMPK‑mediated phosphorylation at Thr177 and Ser538 in mice enhances PGC-1α transcriptional activity, directly linking cardiac bioenergetic stress to the induction of mitochondrial biogenesis in the heart [112].

Beyond phosphorylation, other PTMs fine-tune PGC-1α. Arginine methyltransferase PRMT1 enhances its transcriptional activity by methylating Arg665, Arg667, and Arg669 in the C-terminal region, representing one of the key molecular mechanisms linking nutrient deficiency to cardiac energy metabolism failure and ultimately to the development of cardiomyopathy [113, 114]. PGC-1α recruits O-GlcNAc transferase to modify FoxO transcription factors, integrating nutrient flux into the transcriptional program [115]. In the cardiovascular system, this pathway has been further validated in mouse models of pressure overload‑induced cardiac hypertrophy [116]. SUMOylation at the Lys183 in mice attenuates its coactivator function, whereas deSUMOylation by the SUMO-specific protease SENP1 restores its activity, the dysregulation of which contributes to mitochondrial dysfunction and cardiomyopathy [117, 118]. Acetyltransferases GCN5 and PCAF acetylate and inhibit PGC-1α, whereas the NAD^+^-dependent deacetylase SIRT1 restores its activity through deacetylation [119]. In mice and rats, SIRT1-mediated deacetylation of PGC-1α has been shown to play a protective role in models of HF, diabetic cardiomyopathy, and AS [120, 121]. Furthermore, in rats, GCN5-mediated acetylation of PGC-1α has been directly linked to Angiotensin II-induced vascular hypertrophy [111]. Therefore, restoring a pro-bioenergetic PTM profile on PGC-1α represents a promising therapeutic strategy to enhance mitochondrial content and function in CVDs.

### The regulation of mitochondrial unfolded protein response (UPRᵐᵗ) by PTMs

The UPRᵐᵗ is an adaptive transcriptional program activated upon mitochondrial proteotoxic stress. PTMs play a pivotal role in initiating and modulating this response, determining whether cells restore homeostasis or succumb to death. The key upstream event is the phosphorylation‑driven activation of the ISR. Phosphorylation of eIF2α on serine 51 by kinases such as GCN2 (activated by uncharged tRNAs), PERK (activated by ER-mitochondria crosstalk) and HRI (activated by heme deprivation/oxidative stress) converges to reduce global translation initiation. This attenuation of bulk protein synthesis paradoxically facilitates the preferential translation of specific mRNAs containing upstream open reading frames, most notably the transcription factors ATF4, ATF5, and CHOP [61, 122]. ATF4 and ATF5 serve as master orchestrators of the UPRᵐᵗ by inducing genes that encode mitochondrial chaperones (HSP60, mtHSP70), proteases (ClpP, LONP1), and antioxidant enzymes, thereby expanding the organelle’s protein‑folding and quality control capacity. Although CHOP contributes to the pro‑survival phase, it is ultimately a key executioner of the terminal UPR. Its persistent upregulation triggers apoptosis, and thus establishing a direct molecular link between unresolved mitochondrial stress and RCD. In mouse and rat models of cardiac I/R, this pathway is activated as an early protective mechanism [67, 123]. Furthermore, the nucleocytoplasmic trafficking of ATF5 itself is regulated in response to mitochondrial stress, ensuring signal transduction from the organelle to the nucleus [124].

Beyond the phosphorylation‑centric ISR axis, additional PTMs provide complementary dimensions of UPRᵐᵗ control. For instance, the NAD⁺‑dependent deacetylase SIRT3 directly enhances the folding capacity of mitochondrial chaperones through deacetylation, representing a rapid, transcription‑independent adjustment that reinforces the proteostasis network [125]. Conversely, the duration and intensity of eIF2α phosphorylation are tightly governed by stress‑inducible phosphatases, such as the GADD34/PP1 complex [123], which mediate feedback dephosphorylation. Disruption of this complex as a PTM rheostat can lead to either premature termination or pathological prolongation of the UPRᵐᵗ. In cardiomyocytes subjected to I/R, early GADD34 induction (acute phase of reperfusion) facilitates eIF2α dephosphorylation to restore protein synthesis, whereas delayed or impaired GADD34 expression prolongs the UPRᵐᵗ and shifts the response toward CHOP-mediated apoptosis [126, 127]. Although direct studies on PTM regulation of UPRᵐᵗ in the heart are still emerging, the central role of eIF2α phosphorylation suggests that its manipulation could influence pathophysiological outcomes. Persistent or insufficient UPRᵐᵗ signaling, potentially due to dysregulated PTMs (e.g., altered eIF2α kinase/phosphatase balance), may tip the balance from adaptation to cell death, contributing to the progression from compensated hypertrophy to HF.

In summary, PTMs constitute a sophisticated and dynamic regulatory pattern that governs all aspects of MQC. In the cardiovascular system, this pattern is continuously remodeled in response to physiological and pathological stimuli. Deciphering how specific PTM patterns on MQC proteins are altered across different CVDs is essential for understanding disease pathogenesis. More importantly, this knowledge unveils the modifying enzymes (kinases, phosphatases, acetyltransferases, deubiquitinases) as novel therapeutic targets. The goal is not merely to inhibit or activate a single enzyme, but to strategically modulate the PTM landscape to restore MQC homeostasis and redirect cell fate towards survival in the failing heart.

## PTM regulation of mitochondria in cell death pathways of CVD

mtPTMs are pivotal arbiters of cell fate, critically influencing the initiation and execution of various RCD pathways. They exert control by modulating key mitochondrial properties, including membrane permeability, respiration, ROS generation, and dynamics. Currently, it is well established that mtPTMs can regulate apoptosis, necroptosis, pyroptosis, ferroptosis, and cuproptosis. These specific modes of cell death serve as critical determinants of cardiomyocyte fate in CVD, profoundly influencing pathological progression. Notably, the pathogenic significance of these pathways extends beyond the mere terminal cell clearance. Sub-lethal activation of RCD signaling cascades is increasingly recognized as a potent driver of inflammation, fibrosis, and adverse remodeling even when limited cell death is observed in vivo [128, 129].

### Modulation of apoptosis and necroptosis by mtPTMs in CVD

#### Overview of apoptosis and necroptosis

**Fig. 3:**
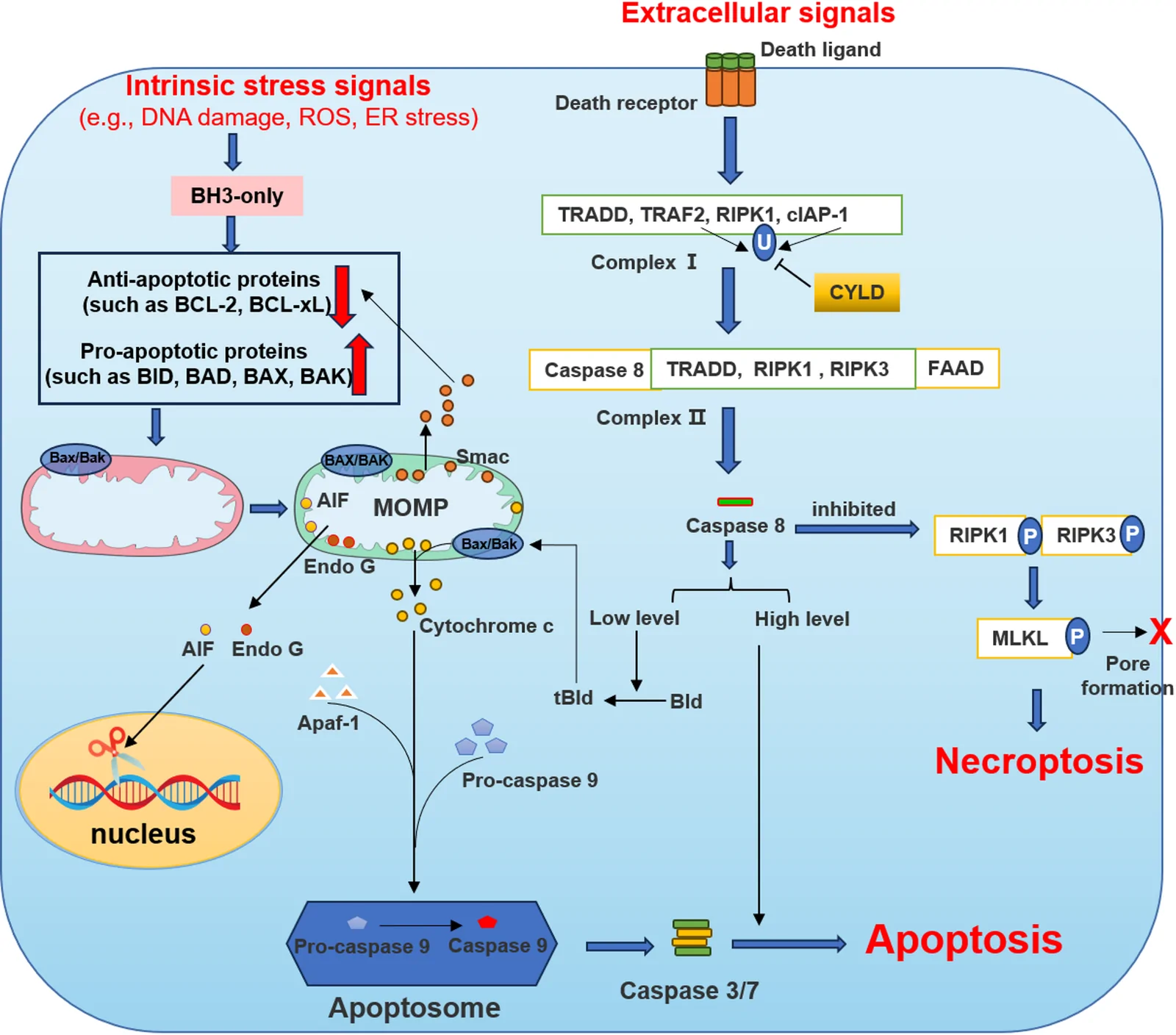
Key pathways of intracellular apoptosis and necroptosis. The intrinsic apoptotic pathway is initiated by intracellular stress signals. This leads to the activation of BH3-only proteins (e.g., BId, BAD, BIM), which inhibit anti-apoptotic proteins (BCL-2, BCL-xL) and activate pro-apoptotic effectors (BAX, BAK). Activated Bax/Bak oligomerize on OMM, inducing MOMP. MOMP causes the release of cytochrome c, SMAC and caspase-independent apoptotic effectors (such as AIF, Endo G) into the cytosol. Cytochrome c, together with Apaf-1 and pro-caspase-9, forms the apoptosome, leading to Caspase-9 activation and subsequent cleavage of executioner Caspases-3/7. SMAC promotes apoptosis by antagonizing inhibitor of apoptosis proteins (IAPs). Endo G directly cleaves nuclear DNA, while AIF recruits other nucleases to induce large-scale DNA fragmentation. The extrinsic apoptotic and necroptotic pathways are triggered by extracellular ligands binding to their respective death receptors. This triggers the sequential assembly of Complex I (containing TRADD, TRAF2, RIPK1, and cIAP1) and Complex II (comprising TRADD, RIPK1, RIPK3, FADD, and Caspase-8). High Caspase-8 activity directly activates Caspases-3/7, whereas low activity results in BId cleavage to tBId, which engages the mitochondrial pathway via BAX/BAK. When Caspase-8 is inhibited, RIPK1 and RIPK3 undergo phosphorylation, leading to MLKL activation. Phosphorylated MLKL oligomerizes and translocates to the plasma membrane. There, its pore assembly disrupt membrane integrity to drive necroptotic cell death

Apoptosis is the first identified and evolutionarily conserved form of programmed cell death (PCD)—a subset of RCD—and serves as the primary mechanism for physiological cell turnover. It is characterized by distinct morphological features, cell shrinkage, chromatin condensation, plasma membrane blebbing, and apoptotic body formation. Its canonical cascades include intrinsic (mitochondrial) pathway and the extrinsic (death receptor) pathway, both of which are executed via caspase activation [130]. A vital event in the intrinsic pathway is mitochondrial outer membrane permeabilization (MOMP), which leads to cytochrome c release and apoptosome formation. Necroptosis, in contrast, is a caspase-independent, inflammatory form of RCD executed through the RIPK1–RIPK3–MLKL signaling axis, culminating in plasma membrane rupture and the release of damage-associated molecular patterns [131]. These pathways are interconnected, for instance, inhibition of Caspase-8 can redirect extrinsic death signaling from apoptosis toward necroptosis. Figure 3 depicts the bifurcation point at the level of Caspase-8 and RIPK1 that determines cell fate between these two death modalities.

#### The regulatory mechanisms of apoptosis and necroptosis by mtPTMs in CVD

In CVD, the fine-tuned balance between cell survival and apoptosis is tightly regulated by PTMs on key BCL-2 family proteins and associated regulators. In mouse models of diabetic cardiomyopathy, AMPK activation triggers a signaling cascade through MAPK8 phosphorylation. This leads to phosphorylation of BCL2 and dissociation of BECN1-BCL2 complex, ultimately restoring autophagy, inhibiting apoptosis, and improving cardiac function [132]. Under cardioprotective signals such as ischemic preconditioning in rodent hearts and neonatal rat cardiomyocytes, AKT- and PKA-mediated phosphorylation of BAD promotes its binding to 14-3-3 proteins, resulting in BAD sequestration and preventing its inhibition of BCL-2/BCL-xL, which effectively suppresses apoptosis [133–135]. In failing human hearts, sustained JNK-mediated phosphorylation enhances the pro-apoptotic activity of BIM and promotes cardiomyocyte apoptosis [136].

In mouse models of myocardial infarction, the balance between pro-survival kinases (AKT/ERK) and the cell cycle-regulated kinase such as CDK1/cyclin B1 in the phosphorylating of Caspase-9 at distinct residues dictates whether cardiomyocytes undergo apoptosis or survival [137, 138]. AKT and ERK phosphorylate Caspase-9 at Ser-196 and Thr-125 in human cancer cell lines and neonatal rat cardiomyocytes, respectively, to disrupt apoptosome assembly or direct inhibit their enzymatic activity, thereby promoting cell survival [139, 140]. In a distinct regulatory context, phosphorylation of Caspase-9 at Thr-125 by CDK1/cyclin B1 paradoxically restrains rather than promotes its pro-apoptotic activity. In mitotic human cells and during G₂/M cell cycle arrest induced by microtubule poisons, this modification sets an elevated threshold for apoptosis activation, thereby protecting cells from death during mitosis despite the presence of pro-apoptotic signals [141].

The phosphorylation and ubiquitination of RIPK1 and RIPK3 critically determine cell fate by directing the canonical RIPK1–RIPK3–MLKL axis toward necroptosis, apoptosis, or survival, as demonstrated in both human cells and rodent models of CVD [142, 143]. Pharmacological inhibition of RIPK1 has been demonstrated to confer cardioprotection in mouse models of MIRI, yet clinical translation remains challenging [144]. Although these kinases primarily reside in the cytoplasm, they can indirectly impact mitochondrial function through modulation of energy metabolism and ROS production. In a mouse model of alcoholic cardiomyopathy, ethanol induces EGR1-mediated upregulation of USP53, which removes K63-linked ubiquitination at RIPK1-K377 to promote its phosphorylation and activate downstream apoptotic and necroptotic pathways, consequently driving disease progression [145].

In various CVDs, mtPTMs significantly influence pathological progression via apoptosis and necroptosis, either improving or exacerbating disease outcomes. Table 1 provides a curated list of key PTMs of mitochondrion-related proteins events on major apoptotic/necroptotic regulators and their specific roles in different CVD models. Moreover, these modifications often engage in cross-regulatory interactions. For instance, DRP1 SUMOylation can influence its phosphorylation status in mouse models of I/R injury [146, 147]. In mouse HF models, SIRT3-mediated deacetylation of CypD not only directly inhibits its activity but may also facilitate its ubiquitination and degradation [148]. Such crosstalk offers novel insights into the regulatory networks governing cell death in CVD.

**Table 1:** Representative PTM events of mitochondrion-related proteins regulating apoptosis and necroptosis in CVDs

Protein	PTM type	Functional impact	Cardiovascular pathological significance	References
BAD	Phosphorylation (Ser155/Ser112)	Inhibits pro-apoptotic activity of Bad	Inhibits apoptosis in neonatal rat cardiomyocytes	[182]
BAD	Phosphorylation	Inhibits apoptosis	Inhibits apoptosis in mouse vascular smooth muscle cells	[183]
DRP1	DeSUMOylation	Promotes apoptosis	Aggravates cardiomyopathy in murine cardiomyocytes	[184]
DRP1	Dephosphorylation (Ser616)	Inhibits apoptosis	Relieves myocardial I/R injury in rats	[185]
DRP1	Dephosphorylation (Ser637)	Promotes apoptosis	Aggravates doxorubicin-induced cardiotoxicity in human and mouse cardiomyocytes	[76]
DRP1	Phosphorylation (Ser656)	Inhibits apoptosis	Stabilizes the myocardial mitochondrial network in rats	[186]
VDAC1	Ubiquitination	Induces autophagy to suppress apoptosis	Alleviates MIRI in cardiomyocyte cell line H9c2	[187, 188]
VDAC1	Phosphorylation (Ser12/Ser103)	Promotes apoptosis	Aggravates apoptosis in human microvascular endothelial cells	[189]
CypD	Ubiquitination	Inhibits mPTP opening and necroptosis	Relieves MIRI in neonatal rat cardiomyocytes and mouse hearts	[69]
CypD	Deacetylation	Inhibits apoptosis	Relieves hypoxia/reoxygenation-induced injury in cardiomyocytes (cell line H9c2)	[190]
OMA1	Ubiquitination	Stabilizes OPA1, reducing apoptosis	Relieves myocardial infarction in mice	[191]
PINK1	Phosphorylation (Ser495)	Activates mitophagy, reducing apoptosis	Relieves HF in transverse aortic constriction-induced mice	[192]
PGC-1α	acetylation	Promoting apoptosis	Aggravates HF in mouse after myocardial infarction	[193]
FOXO3a	Deacetylation	Reduces oxidative damage and apoptosis	Relieves MIRI in rats	[194]
P53	Deacetylation	Reduces ROS production and apoptosis in myocardial cell	Relieves MIRI in isolated rat hearts	[195]

### Modulation of ferroptosis by mtPTMs in CVD

#### Overview of ferroptosis

**Fig. 4:**
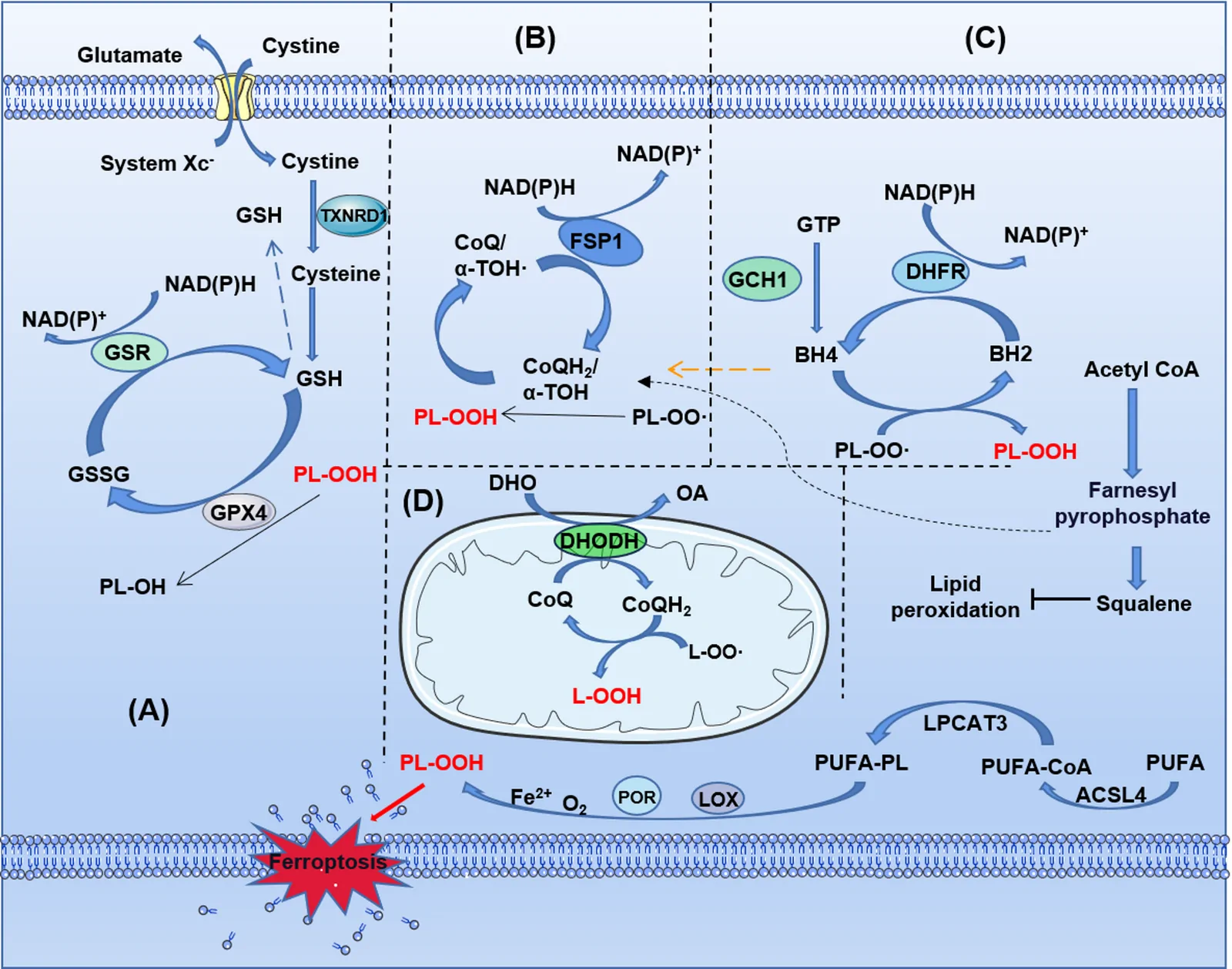
Key regulatory pathways of ferroptosis. Ferroptosis is an iron-dependent form of RCD driven by peroxidation of polyunsaturated fatty acid-containing phospholipids (PUFA-PL). ACSL4 activates PUFAs to PUFA-CoA, which LPCAT3 incorporates into membrane phospholipids. Iron-catalyzed oxidation of PUFA-PL generates phospholipid hydroperoxides (PL-OOH), leading to membrane damage and cell death. **A** System Xc⁻–GSH–GPX4 axis: The plasma membrane transporter System Xc⁻ imports cystine, which is reduced to cysteine through GSH- or thioredoxin reductase 1 (TXNRD1)-dependent pathways. Cysteine is a precursor for GSH synthesis. Using GSH as a cofactor, GPX4 reduces PL-OOH to benign Phospholipid alcohol (PL-OH). Finally, glutathione disulfide reductase (GSR) regenerates GSH from its oxidized form (GSSG) using NAD(P)H as an electron donor. **B** FSP1/CoQ10 system: FSP1 reduces ubiquinone (CoQ) to ubiquinol (CoQH₂) and also regenerates α-tocopherol (α-TOH) from the α-tocopheroxyl radical (α-TOH•). Both CoQH₂ and α-TOH act as radical-trapping antioxidants that suppress lipid peroxidation and inhibit ferroptosis. **C** Squalene and BH4 pathway: GTP cyclohydrolase 1 (GCH1), the rate-limiting enzyme in tetrahydrobiopterin (BH4) synthesis, promotes the synthesis of BH4 and enhances CoQH₂ synthesis and inhibits lipid peroxidation. In parallel, accumulation of squalene can function as a radical scavenger to suppress ferroptosis in specific contexts. **D** Mitochondrial DHODH/CoQ10 system: Dihydroorotate dehydrogenase (DHODH), an inner mitochondrial membrane enzyme, catalyzes the oxidation of dihydroorotate (DHO) to orotate in a coupled reaction that reduces CoQ to CoQH₂. The resulting CoQH₂ acts as a mitochondrial antioxidant that scavenges lipid peroxyl radicals, conferring organelle-specific protection against ferroptosis

Ferroptosis is an iron-dependent, lipid peroxidation-driven mode of RCD formally defined by Dixon et al. in 2012 [149]. It is characterized by distinct morphological alterations in mitochondria, including shrinkage, reduced cristae, and increased membrane density, as well as key biochemical hallmarks such as intracellular iron overload, depletion of glutathione (GSH), inactivation of glutathione peroxidase 4 (GPX4), and accumulation of lipid peroxides [150]. The core regulatory network of ferroptosis primarily involves four major systems: the System Xc⁻–GSH–GPX4 axis, the FSP1/CoQ10 system, the lipid peroxidation inhibition system mediated by squalene and di/tetrahydrobiopterin (BH2/BH4), and the mitochondrial DHODH/CoQ10 system [15, 151]. As illustrated in Fig. 4, these four parallel systems operate across distinct subcellular compartments, with the DHODH/CoQ10 axis providing organelle-specific protection within mitochondria.

#### The regulatory mechanism of ferroptosis by mtPTMs in CVD

mtPTMs are pivotal regulators of ferroptosis in the heart. They exert this role by precisely modulating antioxidant defenses and metabolic enzymes to achieve coordinated control of metabolic and redox homeostasis, thereby presenting promising therapeutic targets for CVD.

At the level of core ferroptosis machinery, the central ferroptosis suppressor GPX4 is destabilized by NEDD4L-mediated ubiquitination, which exacerbates doxorubicin-induced cardiotoxicity in mouse models and cultured cardiomyocytes [152], whereas its stability may also be regulated by SUMOylation in certain contexts [153]. Beyond direct regulators, predominant mitochondrial deacetylase SIRT3 is activated by O-GlcNAcylation at Ser190 in H9C2 cardiomyocytes subjected to simulated I/R, which enhances its ability to deacetylate and activate SOD2. This action suppresses mitochondrial ROS overproduction and maladaptive autophagy, key drivers of ferroptosis, ultimately protecting cardiomyocytes from simulated I/R injury [87]. Additionally, mitochondrial tRNA taurine modification is markedly reduced in ferroptosis-associated myocardial injury in rat, which disrupts mitochondrial translation accuracy and exacerbates cardiac oxidative stress. Notably, taurine supplementation has been shown to improve cardiac tolerance to ferroptosis [154].

At the metabolic level, mitochondrial aldehyde dehydrogenase 2 (ALDH2), a reported endogenous inhibitor of ferroptosis, mitigates high-fat diet-induced obese cardiomyopathy and autophagy deficiency in mouse models by modulating autophagic flux and promoting PGC-1α deacetylation [155]. Moreover, protein modifications directly regulate mitochondrial metabolic enzymes to influence cellular susceptibility to ferroptosis. For instance, SIRT5 confers protection against cardiac I/R injury in mouse hearts by regulating protein succinylation, which restrains succinate dehydrogenase activity and subsequent mitochondrial ROS overproduction to inhibit ferroptosis [156]. Under cardiac energy stress, AMPK activation inhibits ferroptosis by phosphorylation-dependent inhibition of acetyl-CoA carboxylase [157, 158], the fatty acid synthesis rate-limiting enzyme, thereby regulating the cellular PUFA pool that determines ferroptosis sensitivity in mouse cardiomyocytes [158].

### Modulation of pyroptosis by mtPTMs in CVD

#### Overview of pyroptosis

**Fig. 5:**
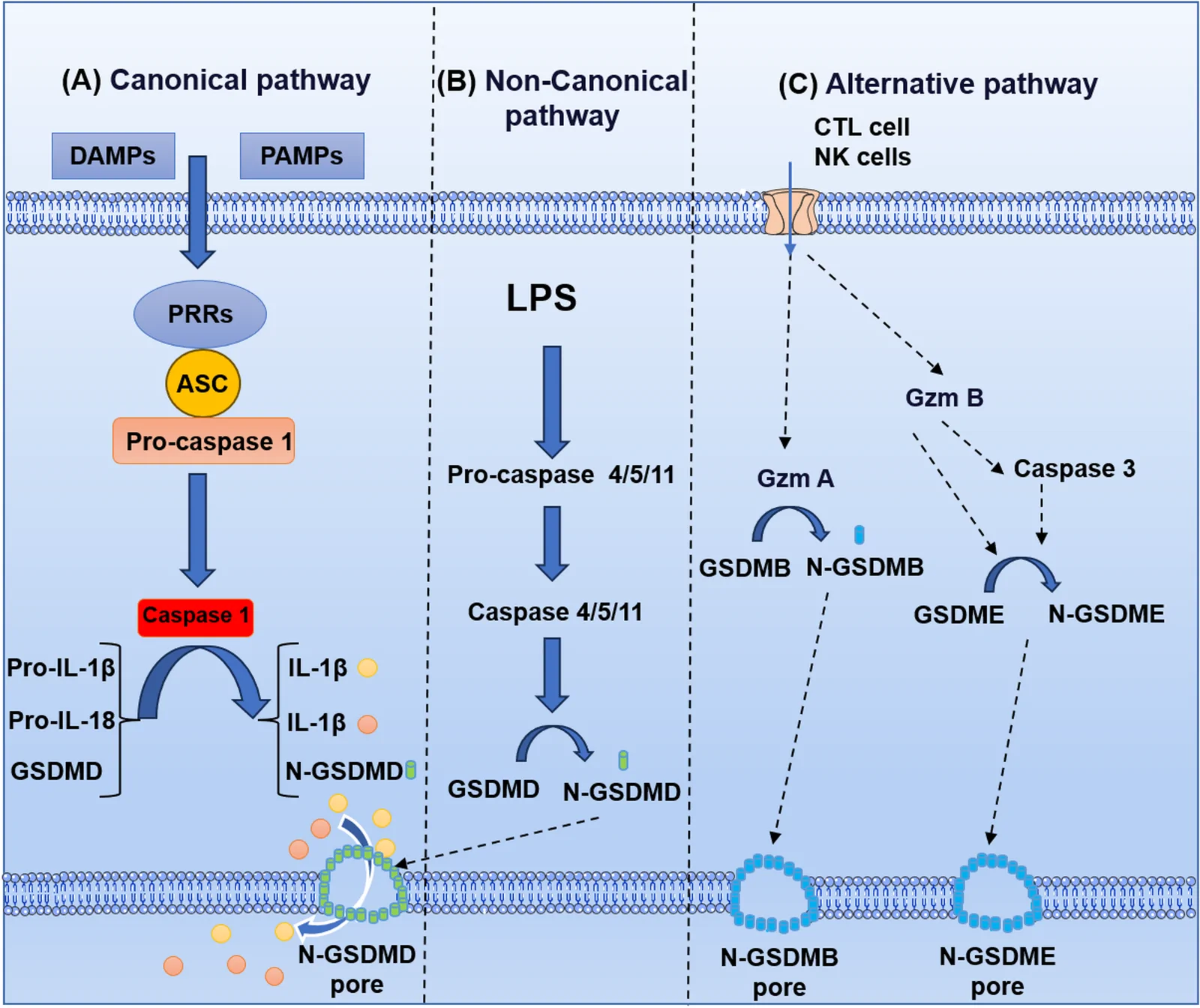
Key signaling pathways in pyroptosis. **A** Canonical inflammasome pathway: Pathogen-associated molecular patterns (PAMPs) or DAMPs are recognized by pattern recognition receptors (PRRs), leading to the recruitment of the adaptor protein ASC and assembly of the inflammasome complex with pro-caspase-1. This triggers inflammasome-mediated Caspase-1 activation, which cleaves pro-IL-1β and pro-IL-18 into their mature forms and processes Gasdermin D (GSDMD) to liberate its N-terminal fragment (N-GSDMD). N-GSDMD then oligomerizes and forms pores in the plasma membrane. **B** Non-canonical pathway: Intracellular lipopolysaccharide (LPS) directly activates Caspase-4/5/11 to induce their oligomerization and activation, leading to GSDMD cleavage and pore formation by GSDMD-N. **C** Granzyme-mediated pathway: Cytotoxic T lymphocytes (CTLs) and natural killer (NK) cells deliver granzymes into target cells. Granzyme A (GzmA) cleaves GSDMB, while granzyme B (GzmB) cleaves GSDME, triggering pyroptosis through their respective pore-forming N-terminal fragments

Pyroptosis is an intensely inflammatory form of RCD [159]. Morphologically, it is characterized by cell swelling and cytoplasmic vacuolization, with relatively minor nuclear alterations. Mechanistically, pyroptosis depends on the activation of Gasdermin family proteins and inflammatory caspases. In the canonical pathway, inflammasome assembly leads to the cleavage of Gasdermin D and the maturation and release of pro-inflammatory cytokines IL-1β and IL-18. Beyond this, pyroptosis can also be activated through non-canonical inflammasome pathways and other Gasdermin family members [160]. Figure 5 illustrates the canonical, non-canonical, and granzyme-mediated pathways converge on Gasdermin cleavage and pore formation.

#### Core mechanisms of pyroptosis by mtPTMs in CVD

In CVD, the initiation and amplification of pyroptosis are critically dependent on the functional state of mitochondria. Mitochondria are not merely energy factories, instead, their proteome, sculpted by a sophisticated network of mtPTMs, directly determines whether a cell maintains homeostasis or undergoes inflammatory death [161]. These mtPTMs serve as core molecular switches in pyroptotic signaling by dynamically regulating MQC and the release of damage-associated molecular patterns (DAMPs). The dysregulation of this precise modification network under pathological stress represents a fundamental mechanism driving inflammatory cell death in the cardiovascular system [162].

The regulatory role of mtPTMs is reflected in their governance over three interconnected aspects: organellar integrity, metabolic state, and signal release. A prime example to modulate mitochondrial integrity is the phosphorylation of DRP1, which promotes its recruitment and oligomerization, driving excessive mitochondrial fission in rat models of MIRI and myocardial infarction [163, 164]. The resulting fragmented organelles are prone to enhanced ROS production and the release of mtDNA, a potent DAMP that activates cytosolic inflammasome sensors and can trigger pyroptosis in cardiomyocytes of diabetic cardiomyopathy mice and in response to various cardiac stresses [165]. Importantly, mtPTMs can also exert protective effects. For instance, in a mouse model of MIRI, VDAC1 phosphorylation serves as a protective mechanism that suppresses GSDME-mediated cardiomyocyte pyroptosis and ameliorates mitochondrial function [166]. Beyond organellar integrity, mtPTMs directly reprogram metabolic and antioxidant pathways. Studies in both animal models and humans have shown elevated acetylation of key metabolic enzymes typically inhibits their activity, leading to cardiac metabolic imbalance and oxidative stress [167]. Conversely, USP14 deubiquitinates and stabilizes the mitochondrial deacetylase SIRT3 in mouse hearts, enhancing its activity to promote antioxidant defense, ultimately attenuating pyroptosis triggered by cardiotoxic agents such as doxorubicin [168].

### Modulation of cuproptosis by mtPTMs in CVD

#### Overview of cuproptosis

**Fig. 6:**
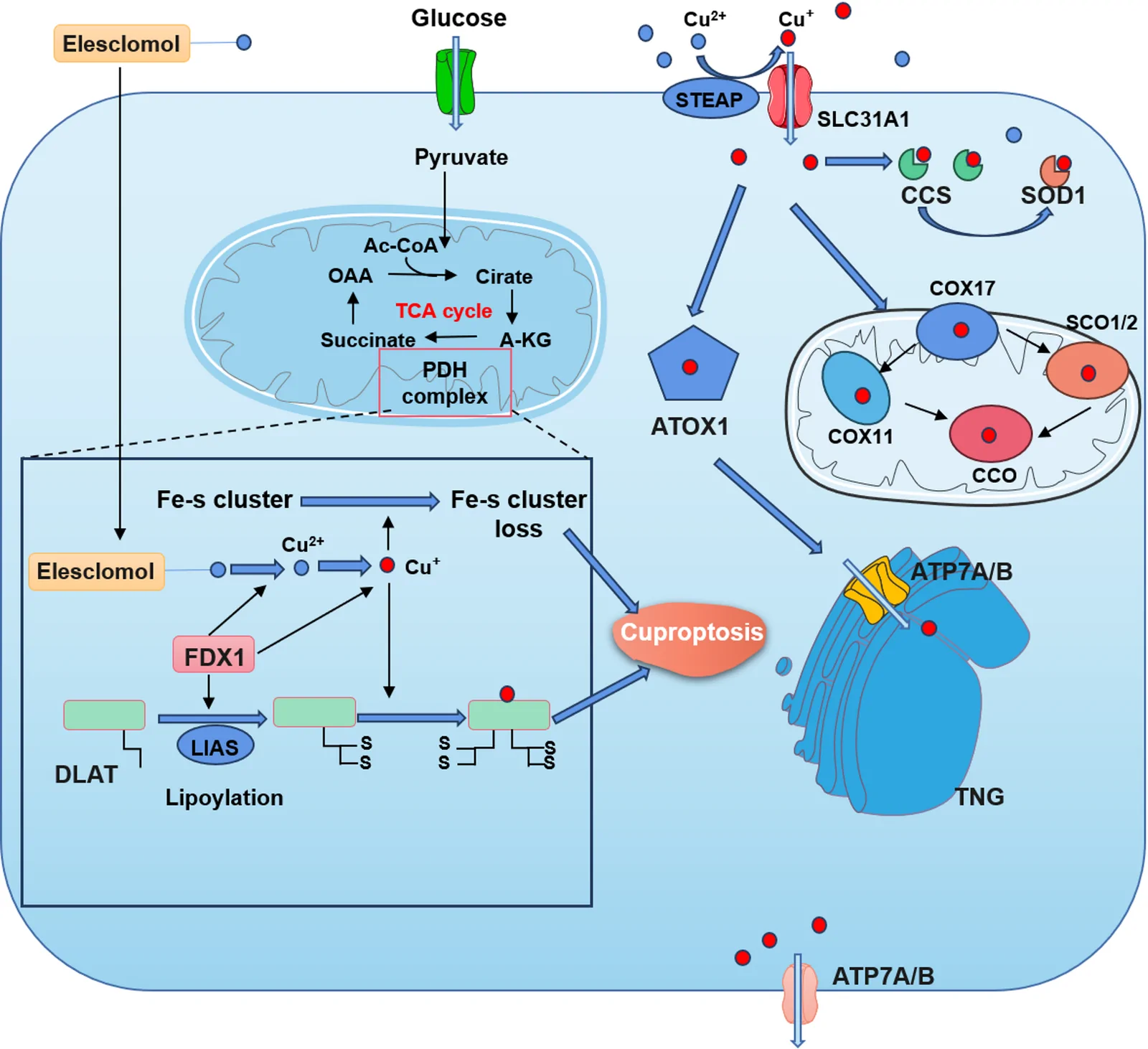
Core pathway of mitochondria-mediated cuproptosis. Extracellular Cu²⁺ is reduced to Cu⁺ by STEAP metalloreductases and imported into the cell via the SLC31A1 transporter. Intracellular Cu⁺ binds to cytosolic chaperones such as CCS and ATOX1, which deliver it to organelles including mitochondria and the endoplasmic reticulum. Within mitochondria, functional cytochrome c oxidase (CCO), which requires copper delivery via COX17, SCO1/2, and COX11 for its assembly, supports mitochondrial respiration, a metabolic prerequisite for cuproptosis. ATOX1 also mediates copper delivery to ATP7A/B in the trans-Golgi network (TGN), activating cuproenzymes in the secretory pathway. Under elevated cytosolic copper levels, ATP7A/B relocalizes from the TGN to promote copper efflux and maintain homeostasis. Copper ionophores such as elesclomol deliver Cu²⁺ into mitochondria, where ferredoxin 1 (FDX1) reduces it to Cu⁺. This Cu⁺ directly binds to lipoyl groups on TCA cycle enzymes such as DLAT, triggering their oligomerization and toxic aggregation. Concurrent Cu⁺ accumulation may also destabilize iron–sulfur (Fe–S) cluster proteins to exacerbate mitochondrial dysfunction. Together, these events culminate in cuproptosis

Cuproptosis is a recently identified type of RCD triggered by excessive copper ion accumulation within mitochondria [169]. Its defining characteristic is a strict dependence on mitochondrial respiration and protein lipoylation. Figure 6 outlines the unique pathway of cuproptosis, highlighting the essential role of FDX1 in reducing Cu²⁺ and facilitating the lipoylation of DLAT, which leads to toxic protein aggregation. This novel pathway operates through a sequential mechanistic cascade [170]. Initially, FDX1 mediates the reduction of cupric ions to cuprous ions and subsequently facilitates the lipoylation of proteins via lipoic acid synthase (LIAS). This process triggers the aggregation of key lipoylated TCA cycle enzymes, such as DLAT and PDHA1. This aggregation in turn leads to the destabilization of iron-sulfur cluster proteins. Critically, cuproptosis operates independently of other known cell death executors, and thus constitutes a unique, metabolically driven form of RCD governed by mitochondrial activity and specific PTMs.

#### The potential mechanism and significance of cuproptosis by mtPTMs in CVD

Emerging evidence underscores the significance of cuproptosis in the pathogenesis of various CVDs. SIRT7 alleviates myocardial fibrosis and cardiac dysfunction in hypertensive rats by activating the YAP/ATP7A signaling axis to enhance copper efflux and suppress cuproptosis in cardiac fibroblasts [171]. In contrast, in a mouse model of transverse aortic constriction-induced pressure overload, SIRT3 downregulation disrupts the interaction between copper transporters and LC3B, impairing copper homeostasis. This leads to intracellular copper accumulation and increased susceptibility to cuproptosis, ultimately accelerating pathological cardiac remodeling under pressure overload [172]. Phillygenin has been shown to protect against MIRI in mice by inhibiting cardiomyocyte cuproptosis through reduction of copper accumulation and suppressing FDX1/LIAS expression [173]. Similarly, hesperetin methyl chalcone alleviates copper deposition by promoting deubiquitination and stabilization of ATP7A, thus ameliorates MIRI in mice [174]. On the other hand, Ub-conjugating enzyme UBE2D3, which is upregulated in mouse models of myocardial infarction, exacerbates cardiomyocyte damage by increasing the expression of FDX1 and SLC31A1 [175]. Moreover, Proprotein Convertase Subtilisin/Kexin Type 9 (PCSK9) directly binds to and stabilizes LIAS, thereby promoting lipoic acid synthesis and subsequent protein lipoylation (e.g., of DLAT), triggering cuproptosis in cardiomyocytes. Preclinical studies indicate that inhibition of PCSK9 by evolocumab (a clinically approved therapy for hypercholesterolemia) disrupts this interaction with LIAS and protects against MIRI in mice [176]. Whether this cardioprotective mechanism translates to humans remains to be determined. These findings not only identify cuproptosis as a novel cell death mechanism in CVD, but also highlight the central role of mtPTMs in regulating copper homeostasis and affecting cell fate.

## mtPTMs as an integrative network governing cell fate decisions in CVD

The preceding sections delineate the regulatory roles of specific mtPTMs in discrete MQC processes and RCD pathways. However, in the complex pathophysiology of CVD, these pathways do not function in isolation. Instead, they constitute a highly interconnected and dynamic network, with mtPTMs acting as the central rheostat. By integrating diverse metabolic and proteotoxic stress signals, mtPTMs ultimately dictate the cellular transition between survival and death.

### Crosstalk and convergence among regulated cell death pathways

While apoptosis, necroptosis, pyroptosis, ferroptosis, and the recently characterized cuproptosis possess unique biochemical triggers and executioners, they converge at critical mitochondrial nodes. This interconnection facilitates signal amplification and modality switching, which can exacerbate myocardial injury during I/R or heart failure. For instance, RIPK1 functions as a molecular switch through its precisely modulated phosphorylation and ubiquitination [177]. Regulated by death receptors and Caspase-8, RIPK1 governs the bifurcating choice between apoptosis and necroptosis through its control over MOMP or mPTP opening. Furthermore, mitochondrial ROS bursts and lipid peroxidation products generated during ferroptosis serve as potent DAMPs that activate the NLRP3 inflammasome, thereby bridging oxidative metabolic damage to inflammatory pyroptosis [178, 179]. Similarly, copper overload induces mitochondrial metabolic collapse and ROS overproduction [170]. Beyond the canonical cuproptosis pathway, this proteotoxic stress can simultaneously trigger intrinsic apoptosis via MOMP or activate the NLRP3-pyroptosis axis, demonstrating a multi-layered death response to heavy metal dyshomeostasis [180].

### mtPTMs as the master conductor of the cell fate network

The functional output of this interconnected network is decisively regulated by dynamic and context-specific mtPTM patterns. In this hierarchical framework, a singular PTM event on a master regulator, such as SIRT3-mediated deacetylation, can resonate across multiple pathways to produce a coherent cellular response. It simultaneously augments mitophagy (via PINK1/Parkin deacetylation), inhibits pathological fission (via OPA1 stabilization), suppresses ferroptosis (via SOD2 activation), and mitigates pyroptosis by preserving mitochondrial structural integrity. On the contrary, aberrant phosphorylation of DRP1 at Ser616 (a key downstream effector modification) initiates a deleterious cascade. It drives excessive mitochondrial fragmentation, impairs mitophagic flux, and lowers the threshold for MOMP, which primes cardiomyocytes for the concurrent engagement of apoptotic, necroptotic, and ferroptotic programs. Critically, ultimate cellular fate is not determined by any single modification, but rather by the integrated landscape of multiple mtPTMs, whose combinatorial interpretation is contingent upon the prevailing pathological context and the underlying metabolic state. Thus, the mtPTMs landscape operates as a coordinated, integrative regulatory framework governing cellular decision-making, rather than as a set of discrete and independent molecular events.

### Intersection of ferroptosis and cuproptosis: a paradigm of mtPTMs-mediated integration

The interplay between ferroptosis and cuproptosis exemplifies the convergence of mtPTMs on shared mitochondrial regulatory hubs, despite their distinct primary triggers. Ferroptosis is driven by iron-dependent lipid peroxidation, whereas cuproptosis involves copper-induced aggregation of lipoylated TCA cycle enzymes [181]. Both pathways intersect at the FDX1-CoQ axis, a vital nexus for mitochondrial redox homeostasis. FDX1 is a bifunctional protein: in cuproptosis, it catalyzes the reduction of Cu²⁺ to the more cytotoxic Cu⁺ species and promotes protein lipoylation; concurrently, it supplies electrons to support CoQ biosynthesis. The reduced form, CoQH₂, is a crucial lipophilic antioxidant and a core component of the mitochondrial DHODH/CoQH₂ axis, the primary defense system against ferroptosis. Therefore, PTMs targeting FDX1 (e.g., phosphorylation or ubiquitination) can simultaneously modulate copper toxicity and the cell’s capacity to quench lipid peroxyl radicals. Moreover, sirtuin (SIRT3, SIRT5) -mediated deacetylation and SUMOylation calibrate the activity of TCA cycle enzymes such as succinate dehydrogenase. This coordinated regulation establishes a shared metabolic‑stress that primes the cell for susceptibility to both ferroptosis and cuproptosis.

### Therapeutic implications of targeting the mtPTMs network

This integrative perspective necessitates a paradigm shift in therapeutic strategy. Instead of targeting a single downstream executioner, future interventions should aim to modulate nodal points within the mtPTMs-based regulatory network to rebalance the entire system. However, these expectations must be tempered by clinical realities. Despite extensive and promising preclinical evidence, no agents specifically targeting a single mtPTM (e.g., a specific DRP1-Ser616 kinase inhibitor) have successfully completed clinical trials in CVD. Major barriers include the achievement of mitochondria-specific delivery, mitigation of on-target toxicity in non-cardiac tissues, and the development of reliable biomarkers to enable appropriate patient stratification and therapeutic selection. For example, in MIRI where cuproptosis and ferroptosis often co-exist, a rational combinatorial preclinical approach could entail enhancing copper efflux through promotion of the deubiquitination and stabilization of the copper transporter ATP7A, alongside reinforcement of the mitochondrial antioxidant capacity via pharmacological activation of the DHODH/CoQH₂ axis. Such strategies aim to restore mitochondrial decision-making integrity by simultaneously alleviating proteotoxic and oxidative stress. Future efforts should prioritize the development of mitochondria-targeted delivery platforms and the integration of patient-specific models to delineate disease-relevant mtPTMs signatures, paving the way for precision cardiac therapeutics.

## Conclusion and perspectives

This review has systematically delineated the central role of mtPTMs as a dynamic molecular code orchestrating MQC and RCD in the cardiovascular system. In the healthy heart, this regulatory network calibrates mitochondrial function to meet high metabolic demands. However, under pathological conditions, dysregulated mtPTMs signatures disrupt MQC homeostasis and sensitize cardiomyocytes to a spectrum of death pathways, driving the progression of various CVDs.

Viewing mtPTMs as an integrative network represents an advance in our understanding of CVD pathogenesis. Introducing a conceptual hierarchy among PTMs separates master regulatory switches from downstream effectors and distinguishes rapid signaling events from longer-term adaptive responses. This hierarchy provides a more nuanced framework for dissecting these complex interactions. The extensive crosstalk between pathways, exemplified by the FDX1-CoQ hub, reveals that cell fate is determined by a coordinated PTM signature rather than isolated events. Consequently, therapeutic strategies must move beyond targeting single molecules or individual pathways toward network-level rebalancing. However, several challenges remain for clinical translation. Elucidating the functional complexity of PTM in vivo is technically demanding, and much of the existing evidence derived from pre-clinical models may not fully recapitulate human pathophysiology. Furthermore, the development of tools for the spatiotemporally precise manipulation of mtPTMs within mitochondria remains in its infancy.

Building upon this integrative network perspective, future research must focus on developing innovative tools to precisely decode and manipulate the mtPTMs landscape. This effort requires the creation of spatiotemporally resolved biosensors for real-time mapping of mtPTMs dynamics within the stressed myocardium. It also necessitates the construction of organelle-specific delivery systems, such as mitochondria-targeted nanoparticles, extracellular vesicles, or peptides, to modulate specific PTM enzymes with high fidelity. Furthermore, establishing human iPSC-derived cardiomyocyte models is essential to validate mtPTMs signatures across diverse patient genetic backgrounds and ultimately facilitate the development of personalized therapeutic strategies.

In conclusion, mtPTMs serve as the master conductors of mitochondrial integrity and cellular fate. By advancing our ability to modulate this intricate molecular network, we can develop a new generation of mechanism-based therapies that address the foundational dysregulations underlying heart failure and other CVDs.

## Supplementary Information

Supplementary Material 1.

## Data Availability

No datasets were generated or analysed during the current study.

## Abbreviations

ALDH2: Aldehyde dehydrogenase 2
AS: Atherosclerosis
CCO: Cytochrome c oxidase
CTLs: Cytotoxic T lymphocytes
CVD: Cardiovascular diseases
DAMPs: Damage-associated molecular patterns
DDX17: DEAD-box helicase 17
DHO: Dihydroorotate
DHODH: Dihydroorotate dehydrogenase
DUBs: Deubiquitinating enzymes
DRP1: Dynamin-related protein 1
FDX1: Ferredoxin 1
Fe–S: Iron–sulfur
GCH1: GTP cyclohydrolase 1
GPX4: Glutathione peroxidase 4
GSDMD: Gasdermin D
GSH: Glutathione
GSR: Glutathione disulfide reductase
GzmA: Granzyme A
GzmB: Granzyme B
HFpEF: Heart failure with preserved ejection fraction
I/R: Ischemia-reperfusion
IAPs: Inhibitor of apoptosis proteins
ISR: Integrated stress response
LPS: Lipopolysaccharide
mtDNA: Mitochondrial DNA
MFN2: Mitofusin-2
MIRI: Myocardial ischemia-reperfusion injury
MOMP: Mitochondrial outer membrane permeabilization
MQC: Mitochondrial quality control
N-GSDMD: N-terminal fragment
NK: Natural killer
OGT: O-GlcNAc transferase
OMM: Outer mitochondrial membrane
OPTN: Optineurin
PAMPs: Pathogen-associated molecular patterns
PCD: Programmed cell death
Proprotein Convertase Subtilisin/Kexin Type 9: PCSK9
PGAM5: Phosphatase phosphoglycerate mutase 5
PKA: Protein kinase A
PL-OH: Phospholipid alcohol
PL-OOH: Phospholipid hydroperoxides
PRRs: Pattern recognition receptors
PTMs: Post-translational modifications
PUFA-PL: Polyunsaturated fatty acid-containing phospholipids
RCD: Regulated cell death
ROS: Reactive oxygen species
TBK1: TANK-binding kinase 1
TCA: Tricarboxylic acid
TGN: Trans-Golgi network
TXNRD1: Thioredoxin reductase 1
UPRᵐᵗ: Mitochondrial unfolded protein response
Ub: Ubiquitin
eNOS: Endothelial nitric oxide synthase
α-TOH: α-tocopherol
α-TOH•: α-tocopheroxyl radical
