# Supplementary material for: Post-translational modifications of mitochondrion-related proteins: integrative orchestrators of cell fate networks in cardiovascular disease
Source: Cell Commun Signal. 2026 Jun 13;24:451. doi: 10.1186/s12964-026-03002-y (PMC13491830; doi:10.1186/s12964-026-03002-y)
Supplement: Supplementary file 1 [file 12964_2026_3002_MOESM1_ESM.pdf]

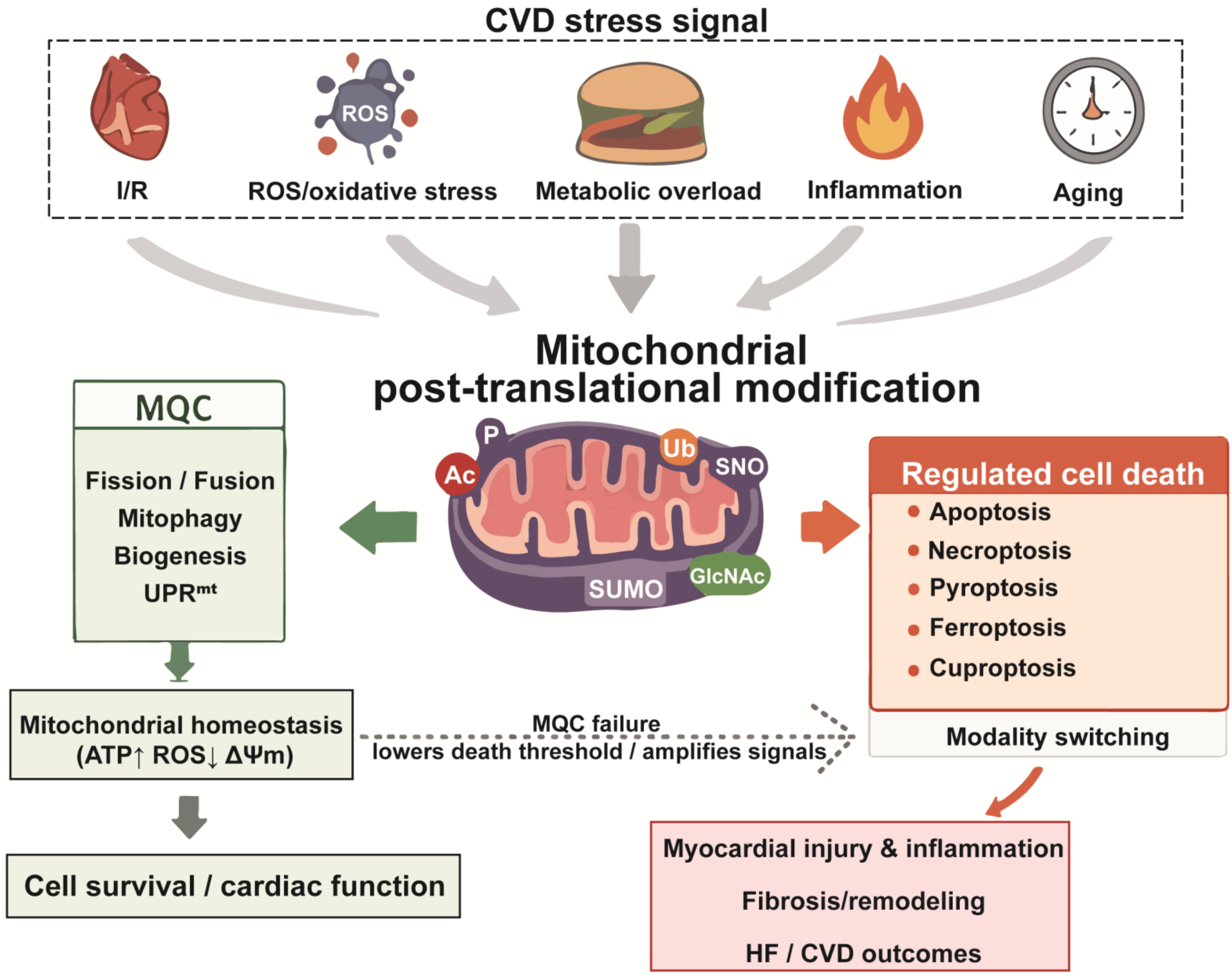

Graphical abstract: Cardiovascular stress reshapes the network of mitochondrial post-translational modifications (mtPTMs), which functions as a molecular modulator coordinating mitochondrial quality control (fission/fusion, mitophagy, biogenesis and UPR^{mt}). Balanced mtPTMs maintain mitochondrial homeostasis (ATP production, restrained ROS and preserved membrane potential), whereas mtPTM network disruption impairs MQC, amplifies mitochondrial damage signals and lowers the death threshold. This promotes crosstalk and switching among apoptosis, necroptosis, pyroptosis, ferroptosis and cuproptosis, ultimately driving myocardial injury, inflammation, adverse remodeling and heart failure.
